# Predicting Phenotypic Polymyxin Resistance in Klebsiella pneumoniae through Machine Learning Analysis of Genomic Data

**DOI:** 10.1128/mSystems.00656-19

**Published:** 2020-05-26

**Authors:** Nenad Macesic, Oliver J. Bear Don’t Walk, Itsik Pe’er, Nicholas P. Tatonetti, Anton Y. Peleg, Anne-Catrin Uhlemann

**Affiliations:** a Division of Infectious Diseases, Columbia University Irving Medical Center, New York, New York, USA; b Department of Infectious Diseases, The Alfred Hospital and Central Clinical School, Monash University, Melbourne, Australia; c Department of Biomedical Informatics, Columbia University, New York, New York, USA; d Department of Computer Science, Columbia University, New York, New York, USA; e Infection and Immunity Program, Monash Biomedicine Discovery Institute, Department of Microbiology, Monash University, Clayton, Victoria, Australia; f Microbiome & Pathogen Genomics Core, Columbia University Irving Medical Center, New York, New York, USA; Agricultural Biotechnology Research Center

**Keywords:** genotype, phenotype, prediction, antimicrobial resistance, machine learning

## Abstract

Polymyxins are used as treatments of last resort for Gram-negative bacterial infections. Their increased use has led to concerns about emerging polymyxin resistance (PR). Phenotypic polymyxin susceptibility testing is resource intensive and difficult to perform accurately. The complex polygenic nature of PR and our incomplete understanding of its genetic basis make it difficult to predict PR using detection of resistance determinants. We therefore applied machine learning (ML) to whole-genome sequencing data from >600 Klebsiella pneumoniae clonal group 258 (CG258) genomes to predict phenotypic PR. Using a reference-based representation of genomic data with ML outperformed a rule-based approach that detected variants in known PR genes (area under receiver-operator curve [AUROC], 0.894 versus 0.791, *P = *0.006). We noted modest increases in performance by using a bacterial genome-wide association study to filter relevant genomic features and by integrating clinical data in the form of prior polymyxin exposure. Conversely, reference-free representation of genomic data as k-mers was associated with decreased performance (AUROC, 0.692 versus 0.894, *P = *0.015). When ML models were interpreted to extract genomic features, six of seven known PR genes were correctly identified by models without prior programming and several genes involved in stress responses and maintenance of the cell membrane were identified as potential novel determinants of PR. These findings are a proof of concept that whole-genome sequencing data can accurately predict PR in K. pneumoniae CG258 and may be applicable to other forms of complex antimicrobial resistance.

**IMPORTANCE** Polymyxins are last-resort antibiotics used to treat highly resistant Gram-negative bacteria. There are increasing reports of polymyxin resistance emerging, raising concerns of a postantibiotic era. Polymyxin resistance is therefore a significant public health threat, but current phenotypic methods for detection are difficult and time-consuming to perform. There have been increasing efforts to use whole-genome sequencing for detection of antibiotic resistance, but this has been difficult to apply to polymyxin resistance because of its complex polygenic nature. The significance of our research is that we successfully applied machine learning methods to predict polymyxin resistance in Klebsiella pneumoniae clonal group 258, a common health care-associated and multidrug-resistant pathogen. Our findings highlight that machine learning can be successfully applied even in complex forms of antibiotic resistance and represent a significant contribution to the literature that could be used to predict resistance in other bacteria and to other antibiotics.

## Abstract (precis)

Polymyxins are last-resort antibiotics used to treat highly resistant Gram-negative bacteria. There are increasing reports of polymyxin resistance emerging, raising concerns of a postantibiotic era. Polymyxin resistance is therefore a significant public health threat, but current phenotypic methods for detection are difficult and time-consuming to perform. There have been increasing efforts to use whole-genome sequencing for detection of antibiotic resistance, but this has been difficult to apply to polymyxin resistance because of its complex polygenic nature. The significance of our research is that we successfully applied machine learning methods to predict polymyxin resistance in Klebsiella pneumoniae clonal group 258, a common health care-associated and multidrug-resistant pathogen. Our findings highlight that machine learning can be successfully applied even in complex forms of antibiotic resistance and represent a significant contribution to the literature that could be used to predict resistance in other bacteria and to other antibiotics.

## INTRODUCTION

Carbapenem resistance in *Enterobacterales* (CRE) is a global health challenge that threatens many medical advances. Due to the lack of antimicrobial options for treating CRE infections, polymyxins (including colistin and polymyxin B) have been revived despite their toxicity and are widely used as treatments of last resort (1). Polymyxin resistance (PR) has now become a growing concern and may critically impair our ability to combat CRE infections. Epidemiological studies have indicated PR rates ranging from 5 to >40% in CRE, with Klebsiella pneumoniae accounting for the majority (1).

While improving the diagnosis and treatment of PR infections is an urgent priority, there are several challenges. Phenotypic polymyxin susceptibility testing is resource intensive and difficult to perform accurately (2). Broth microdilution (BMD) testing is recommended by the CLSI and EUCAST but is often available only in reference laboratories. Many clinical laboratories have traditionally relied on gradient diffusion methods such as the Etest, but significant concerns about the accuracy of these methods have been raised (3, 4). Our understanding of the genetic basis of PR also remains limited. Several important genetic loci of PR have been identified in multiple studies (*crrAB*, *mgrB*, *phoPQ*, *pmrAB*) (5–11) and will henceforth be referred to as PR canonical genes. However, PR is noted in isolates not carrying mutations or disruptions in these canonical genes and may result from mutations in multiple genes (12). Conversely, some susceptible isolates have mutations in canonical genes but do not exhibit PR, raising the possibility of variants that do not cause resistance or compensatory mutations. This makes PR a challenging polygenic trait to diagnose and hampers efforts at rapid molecular detection of PR.

With increasing availability of bacterial whole-genome sequencing (WGS) data, there has been active investigation into using these data for genotype-phenotype prediction of antimicrobial susceptibility testing (AST). This was initially in the form of rule-based approaches that would predict susceptibility through detection of known resistance determinants (e.g., beta-lactamase genes) or known resistance mutations in housekeeping genes (e.g., *rpoB* conferring rifampin resistance in Staphylococcus aureus) (13). The performance of these approaches has varied depending on the organism and antimicrobial tested, but multiple limitations remain. First, the approach assumes that all determinants of resistance are known and is therefore unable to detect previously uncharacterized determinants. Second, these rule-based models struggle to account for complex interactions between variants in multiple loci. In order to move beyond these limitations, machine learning (ML) methods have been used to predict antimicrobial susceptibility (14–17). Given the incomplete identification of contributing PR mutations and the possible polygenic nature of PR, we hypothesize that ML approaches may be well suited to AST genotype-phenotype prediction in this setting (18) and may ultimately be used to help identify isolates for confirmatory phenotypic testing.

We therefore aimed to use ML for genotype-phenotype prediction of PR in K. pneumoniae clonal group 258 (CG258), as this remains the main CRE clone in North America and a key CRE clone globally (19). We aimed to do this using both a reference-based approach that relied on variant calling and insertion sequence (IS) detection and a reference-free approach using detection of k-mers. We compared these ML approaches to a simple rule-based model that relies on detection of variants in canonical PR genes. Finally, we hypothesized that the ML models could be interpreted to confirm biological plausibility and elucidate novel genetic determinants of PR.

## RESULTS

**FIG 1:**
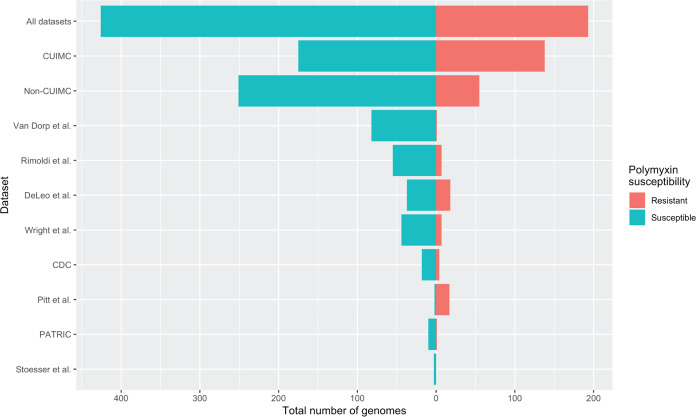
Summary of input data sets according to polymyxin resistance. Histograms show the relative distribution of polymyxin resistance across all genomes, Columbia University Irving Medical Center (CUIMC) genomes only, non-CUIMC genomes only, and then individual publicly available data sets that formed non-CUIMC genomes. For further information regarding individual genomes, see Data Set S1, sheet 1, in the supplemental material.

**FIG 2:**
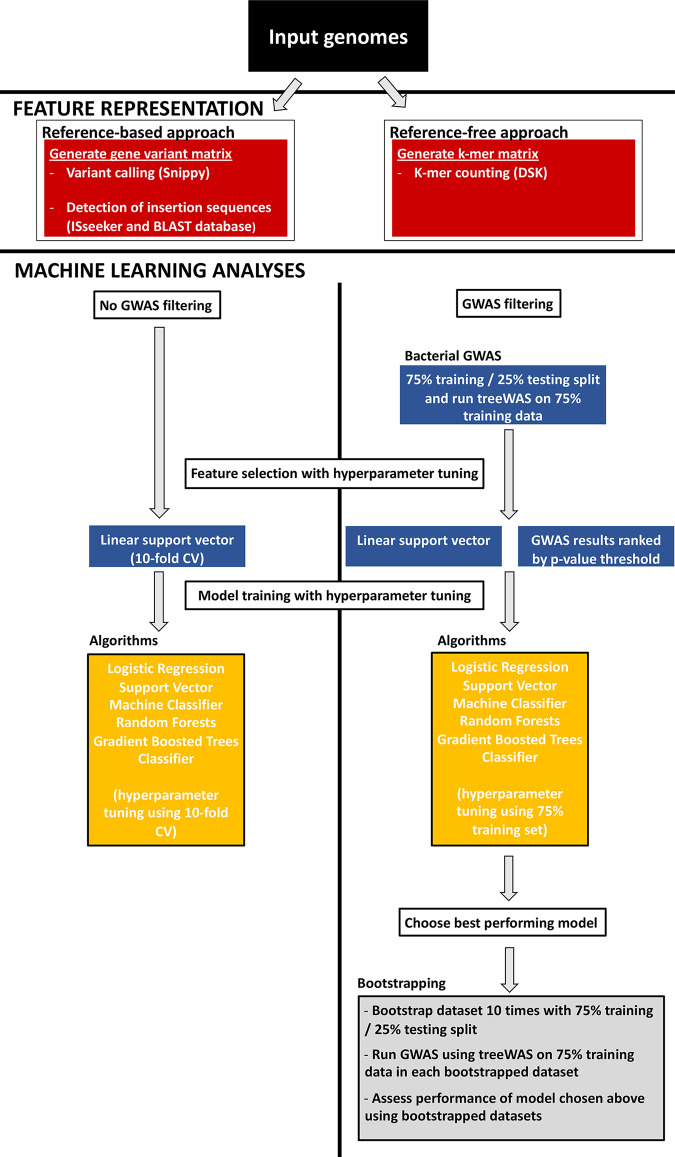
Schematic of polymyxin susceptibility genotype-phenotype prediction using machine learning. Publicly available genomes and genomes from Columbia University Irving Medical Center were processed using either a reference-based or a reference-free approach in order to generate a binary matrix. The binary matrix represented either a gene variant matrix of coding regions in the genome (reference-based approach) or individual k-mers (reference-free approach). For machine learning analyses, two approaches were used. In the non-genome-wide association study (non-GWAS) approach, a scikit-learn pipeline was implemented, which included a further feature selection step using a support vector classifier and then comparison between four algorithms with 10-fold cross-validation (CV) for hyperparameter tuning. For the GWAS filtering approach, further feature selection was performed by integrating the results of a bacterial GWAS to prioritize genes. Model training was performed with hyperparameter tuning using the 75% training set, with the same four algorithms evaluated. The best-performing model was chosen, and the data were bootstrapped 10 times to assess model performance, with GWAS performed on the 75% training split of each data set. Specific tools used during the workflow are noted in the figure.

We analyzed 619 previously sequenced K. pneumoniae CG258 genomes (10–12, 20–25). To examine the impact of groups of input genomes on model performance, we conducted analyses on three different data sets: first, on all available genomes (193/619 genomes with PR; 31%) and then on two subsets of genomes according to their origins (Columbia University Irving Medical Center [CUIMC] or non-CUIMC, 138/313 [44%] and 55/306 [22%] genomes with PR, respectively). This was done due to differing degrees of clonal relatedness according to the data set used and differing rates of PR, with CUIMC genomes having a high degree of clonal relatedness (with some being serial isolates collected from single patients), as well as higher rates of PR (see Fig. S1 in the supplemental material for phylogeny). The data sets and their polymyxin susceptibility are summarized in Fig. 1, including individual non-CUIMC data sets. Full genome details are included in Data Set S1, sheet 1. The study workflow is summarized in Fig. 2. Area under receiver-operator curve (AUROC) was used as the key performance metric, as it is a measure of the trade-off between the true positive rate and the false-positive rate for various decision-making thresholds.

10.1128/mSystems.00656-19.1 FIG S1 Phylogeny of sequences from CUIMC and other data sets. Differing degrees of clonal relatedness according to data set used and differing rates of PR, with CUIMC genomes having a high degree of clonal relatedness and higher rates of PR. Download FIG S1, JPG file, 0.1 MB. Copyright © 2020 Macesic et al. 2020 Macesic et al. This content is distributed under the terms of the Creative Commons Attribution 4.0 International license.

10.1128/mSystems.00656-19.2 DATA SET S1 Sheet 1, details of genomes included in study for machine learning analyses; sheet 2, summary of number of genomes and features in each data set; sheet 3, summary of number of variants in canonical polymyxin resistance genes in each data set; sheet 4, comparison between data sets of mean performance across different metrics with ANOVA; sheet 5, results of genome-wide association study of polymyxin resistance using Columbia University Irving Medical Center genomes; sheet 6, results of genome-wide association study of polymyxin resistance using publicly available genomes; sheet 7, results of genome-wide association study of polymyxin resistance using all included genomes; sheet 8, performance of machine learning analyses for polymyxin resistance prediction using KOVER: sheet 9, comparison of reference-free approaches versus reference-free approaches with genome-wide association study filtering for prediction of polymyxin resistance: sheet 10, comparison of reference-based approaches versus reference-free approaches with genome-wide association study filtering for prediction of polymyxin resistance; sheet 11, genes ranked by relative feature importance in machine learning models for polymyxin resistance prediction. Download Data Set S1, XLSX file, 0.1 MB. Copyright © 2020 Macesic et al. 2020 Macesic et al. This content is distributed under the terms of the Creative Commons Attribution 4.0 International license.

### Machine learning approaches outperform rule-based approach for prediction of polymyxin resistance.

For our reference-based analyses, we created a binary matrix, with genomes as rows and coding regions as columns (referred to as “instances” and “features,” respectively, in ML literature), with the presence of single nucleotide variants (SNVs) or IS elements in coding regions relative to a CG258 reference genome being recorded, as described previously (12) (Data Set S1, sheet 2). This matrix was then used as an input for a simple rule-based approach which classified isolates as PR if there was a variant present in any of the canonical PR genes (*crrAB*, *mgrB*, *pmrAB*, *phoPQ*) (5–11) (Data Set S1, sheet 3). This approach had modest performance, resulting in an AUROC of 0.717 to 0.832, depending on input genomes (Table 1).

**TABLE 1:** Comparison of rule-based and reference-based approaches for prediction of PR^*a*^

Genomes used	Metric	Rule-based value	Ref-based value (95% CI) using best- performing algorithm indicated	Ref-based value with GWAS (95% CI) using best-performing algorithm indicated	*P* value (ref-based vs rule-based)	P value (GWAS vs rule-based)
			Random forest	Random forest		
CUIMC	AUROC	0.832	0.885 (0.849, 0.92)	0.893 (0.864, 0.922)	0.014*	0.004*
	bACC	0.832	0.789 (0.751, 0.827)	0.841 (0.82, 0.862)	0.049^+^	0.262
	Accuracy	0.821	0.796 (0.762, 0.83)	0.854 (0.83, 0.878)	0.185	0.052
	F1	0.819	0.755 (0.701, 0.809)	0.816 (0.793, 0.84)	0.027^+^	0.919
	Precision	0.738	0.799 (0.739, 0.859)	0.881 (0.848, 0.914)	0.037*	0.006*
	Recall	0.92	0.733 (0.635, 0.831)	0.763 (0.725, 0.801)	0.008^+^	0.006^+^
						
			GBTC	SVC		
Non-CUIMC	AUROC	0.717	0.933 (0.884, 0.982)	0.933 (0.888, 0.979)	0.006*	0.002*
	bACC	0.717	0.753 (0.654, 0.853)	0.82 (0.76, 0.881)	0.415	0.006*
	Accuracy	0.699	0.873 (0.822, 0.925)	0.917 (0.894, 0.94)	0.006*	0.006*
	F1	0.471	0.59 (0.395, 0.785)	0.729 (0.648, 0.81)	0.185	0.002*
	Precision	0.345	0.711 (0.473, 0.949)	0.832 (0.759, 0.905)	0.018*	0.006*
	Recall	0.745	0.57 (0.362, 0.778)	0.669 (0.547, 0.791)	0.184	0.262
						
			GBTC	SVC		
All	AUROC	0.791	0.894 (0.838, 0.95)	0.931 (0.915, 0.947)	0.006*	0.002*
	bACC	0.791	0.784 (0.73, 0.838)	0.801 (0.776, 0.827)	0.61	0.375
	Accuracy	0.761	0.827 (0.78, 0.874)	0.864 (0.84, 0.888)	0.019*	0.006*
	F1	0.694	0.702 (0.623, 0.781)	0.741 (0.702, 0.779)	0.76	0.02*
	Precision	0.577	0.8 (0.675, 0.926)	0.889 (0.846, 0.932)	0.011*	0.006*
	Recall	0.87	0.668 (0.549, 0.788)	0.638 (0.591, 0.686)	0.006^+^	0.002^+^

a *, statistical significance with a *P* value of <0.05 in favor of machine-learning approaches; +, statistical significance with a *P* value of <0.05 in favor of rule-based approaches. Abbreviations: PR, polymyxin resistance; AUROC, area under receiver-operator curve; bACC, balanced accuracy; CI, confidence interval; CUIMC, Columbia University Irving Medical Center; GBTC, gradient boosted trees classifier; GWAS, genome-wide association study; ref, reference; SVC, support vector machine classifier.

**FIG 3:**
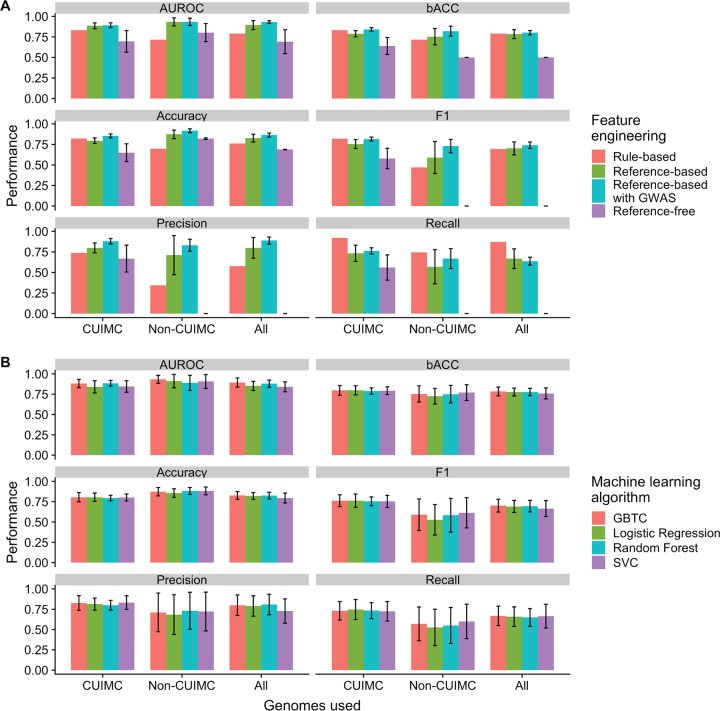
Impact of feature engineering approach and machine learning algorithm on performance of machine learning models for polymyxin resistance prediction. Mean performance with 95% confidence intervals is shown across different performance metrics. The algorithms used were those that achieved the highest area under receiver-operator curve and can be found in Table 3. Histograms show how performance is impacted by the feature engineering approach (A) and the choice of machine learning algorithm (B). Abbreviations: AUROC, area under receiver-operator curve; bACC, balanced accuracy; CUIMC, Columbia University Irving Medical Center; GBTC, gradient boosted trees classifier; GWAS, genome-wide association study; SVC, support vector machine classifier.

We then used the same matrix as an input for ML analyses with four different ML algorithms: logistic regression, random forest, support vector machine classifier (SVC), and gradient-boosted trees classifier (GBTC), as implemented in scikit-learn (26) (code is available on GitHub at https://github.com/crowegian/AMR_ML). The best-performing algorithms achieved mean AUROC of 0.885 to 0.933 (according to input genomes used), and results for these algorithms are shown in Fig. 3A and Table 1. A significant difference in mean performance when different input genome data sets were compared across metrics was noted in accuracy (analysis of variance [ANOVA] *P = *0.0345) (Data Set S1, sheet 4) but not in any other metrics. The reference-based ML analyses achieved significantly higher AUROC than the rule-based approach (*P = *0.014 for CUIMC genomes, *P = *0.006 for non-CUIMC genomes, and *P = *0.006 for all genomes) For other metrics, ML approaches generally performed as well as or better than the rule-based approach, with the exception of recall (Table 1). However, for CUIMC genomes, worse performance was also noted for balanced accuracy and F1 score (Table 1).

### Choice of machine learning algorithm did not impact performance across different input genomes and performance metrics.

The ML algorithms used (logistic regression, random forest, SVC, and GBTC) differ substantially in methodology. To assess whether the choice of ML algorithm impacted performance and whether there was an optimal algorithm, we compared ML algorithms according to input genomes and different performance metrics (Fig. 3B). No significant differences in performance were noted between algorithms, regardless of the input genomes and performance metric used.

### Impact of feature engineering with GWAS filtering and polymyxin exposure data on performance of ML-based prediction.

A key challenge in AST genotype-phenotype prediction is the sparsity of the input genomic data sets due to relatively few genomes in data sets compared to the number of genomic features (14). Appropriate feature selection prior to training ML algorithms is a potential solution to this problem. We hypothesized that conducting a bacterial genome-wide association study (GWAS) as a filtering procedure for selecting the most important features by *P* value would improve the performance of ML models. We therefore conducted a bacterial GWAS using the R package treeWAS in the simultaneous mode (27), which enables correction for population structure. The results of the GWAS are shown in Data Set S1, sheets 5 to 7, according to the input genomes used. Using these results to prioritize genes resulted in a mean increase of 5.3% in performance when all performance metrics were considered (range, −3% to 13.9%), but this moderate rise was not statistically significant for most metrics when standard results were compared with those using GWAS filtering individually (Table 2 and Fig. 3A). There was less variability in model performance when GWAS filtering was applied (Tables 1 and 2).

**TABLE 2:** Comparison of different feature engineering approaches on performance of ML prediction of PR^*a*^

Genomes used	Metric	Ref-based value (95% CI)^*b*^	Ref-based value with GWAS (95% CI)*b*	Ref-free value (95% CI)^*b*^	Ref-based value with polymyxin exposure data (95% CI)^*b*^	*P* value (GWAS vs ref based)	*P* value (ref free vs ref based)	*P* value (poly exposure vs ref based)
		Random forest	Random forest	SVC	GBTC			
CUIMC	AUROC	0.885 (0.849, 0.92)	0.893 (0.864, 0.922)	0.696 (0.564, 0.828)	0.923 (0.88, 0.965)	0.571	0.241	0.104
	bACC	0.789 (0.751, 0.827)	0.841 (0.82, 0.862)	0.64 (0.536, 0.743)	0.796 (0.714, 0.879)	0.026*	0.226	0.544
	Accuracy	0.796 (0.762, 0.83)	0.854 (0.83, 0.878)	0.649 (0.541, 0.758)	0.804 (0.716, 0.892)	0.009*	0.91	0.342
	F1	0.755 (0.701, 0.809)	0.816 (0.793, 0.84)	0.579 (0.454, 0.704)	0.768 (0.685, 0.85)	0.045*	0.734	0.733
	Precision	0.799 (0.739, 0.859)	0.881 (0.848, 0.914)	0.67 (0.506, 0.833)	0.866 (0.737, 0.996)	0.006*	0.023*	0.085
	Recall	0.733 (0.635, 0.831)	0.763 (0.725, 0.801)	0.56 (0.407, 0.714)	0.732 (0.607, 0.857)	1	0.011*	0.879
								
		GBTC	SVC	SVC				
Non-CUIMC	AUROC	0.933 (0.884, 0.982)	0.933 (0.888, 0.979)	0.803 (0.692, 0.913)		0.85	0.677	
	bACC	0.753 (0.654, 0.853)	0.82 (0.76, 0.881)	0.5 (0.5, 0.5)		0.427	0.089	
	Accuracy	0.873 (0.822, 0.925)	0.917 (0.894, 0.94)	0.82 (0.811, 0.83)		0.185	0.005*	
	F1	0.59 (0.395, 0.785)	0.729 (0.648, 0.81)	0 (0, 0)		0.345	0.623	
	Precision	0.711 (0.473, 0.949)	0.832 (0.759, 0.905)	0 (0, 0)		0.703	0.569	
	Recall	0.57 (0.362, 0.778)	0.669 (0.547, 0.791)	0 (0, 0)		0.88	0*	
								
		GBTC	SVC	GBTC				
All	AUROC	0.894 (0.838, 0.95)	0.931 (0.915, 0.947)	0.692 (0.546, 0.838)		0.19	0.015*	
	bACC	0.784 (0.73, 0.838)	0.801 (0.776, 0.827)	0.5 (0.5, 0.5)		0.473	0.006*	
	Accuracy	0.827 (0.78, 0.874)	0.864 (0.84, 0.888)	0.688 (0.685, 0.691)		0.162	0.045*	
	F1	0.702 (0.623, 0.781)	0.741 (0.702, 0.779)	0 (0, 0)		0.384	0.003*	
	Precision	0.8 (0.675, 0.926)	0.889 (0.846, 0.932)	0 (0, 0)		0.363	0.472	
	Recall	0.668 (0.549, 0.788)	0.638 (0.591, 0.686)	0 (0, 0)		0.344	0.064	

a *, statistical significance with a *P* value of <0.05. Abbreviations: ML, machine learning; PR, polymyxin resistance; AUROC, area under receiver-operator curve; bACC, balanced accuracy; CI, confidence interval; CUIMC, Columbia University Irving Medical Center; GWAS, genome-wide association study; ref, reference.

*^b^*Using the best-performing algorithm indicated.

The widespread availability of electronic medical records has made it possible to rapidly extract clinical data for use in ML approaches (28). We wanted to assess if integrating clinical data would improve performance compared to that of ML models built only with genomic data. Polymyxin exposure has been recognized as a factor contributing to emergence of PR (1, 12, 29). Data regarding polymyxin exposure in patients prior to culture of K. pneumoniae isolates were available for CUIMC genomes (12). This was used to create an additional binary feature of polymyxin exposure, added to the genome binary matrix, and new ML models were trained as per the reference-based approach. Modest increases in all metrics except recall were noted. Integration of polymyxin exposure data resulted in the best-performing model compared with reference-based and GWAS filtering approaches (AUROC, 0.923 versus 0.885 and 0.893, respectively) (Table 2). However, these increases did not reach statistical significance.

### Reference-based approaches outperform reference-free approaches.

Reference-based approaches for creating a genomic feature matrix for ML have inherent limitations. Bacteria such as K. pneumoniae with a large accessory genome pose a particular problem, as a reference-based approach cannot evaluate novel genomic content present in the test genomes but lacking in the reference (15). Use of reference-free approaches, such as the use of k-mers (strings of nucleotides of *k* length) as inputs, may therefore be an attractive solution to overcome this limitation. Furthermore, being able to use reference-free approaches may potentially expand the available data set by including a more diverse collection of genomes and thus improve the performance of ML algorithms.

We tested this hypothesis by generating a reference-free binary matrix based on k-mer profiles from the DSK k-mer counting software (30) using k-mers of 31 nucleotides. This approach resulted in much higher numbers of features being incorporated than in reference-based data sets (415,373 versus 3,054 features for all genomes, 124,954 versus 2,278 features for CUIMC genomes, and 348,157 versus 2,350 features for non-CUIMC genomes, respectively). This matrix was then used as an input into the same ML pipeline as described previously. Despite only focusing on chromosomal changes relative to the reference, reference-based ML models had a higher mean performance than reference-free models across all metrics in all data sets (Table 2), reaching statistical significance when all genomes were used. The reference-free models for the data sets containing all genomes and non-CUIMC genomes predicted the same phenotype for all samples (Table 2).

In order to assess whether these findings may have been due to the ML algorithms in our pipeline not being specifically designed for k-mer data inputs, we used the same binary matrices as inputs in the KOVER package (31). This was developed for the sparse data sets seen when k-mers are used in AST genotype-phenotype prediction and uses either the set covering machine (SCM) algorithm or the classification and regression trees (CART) algorithm. However, performance in KOVER was similar to that of our pipeline using both SCM and CART (AUROC, 0.525 to 0.770) (Data Set S1, sheet 8).

We also attempted to use GWAS filtering on reference-free data sets. We were able to complete analyses for CUIMC and non-CUIMC genomes but exceeded computing capacity for analyses on all genomes. Similar to the effect of GWAS filtering on reference-based data sets, we saw modest improvements in performance that did not reach statistical significance (AUROC, 0.761 versus 0.696 [*P = *0.300] and 0.829 versus 0.803 [*P = *0.627] for CUIMC and non-CUIMC genomes, respectively) (Data Set S1, sheet 9). Despite this modest increase, performance using GWAS filtering for reference-free data sets was lower than for reference-based approaches without GWAS filtering (AUROC, 0.885 versus 0.761 [*P < *0.001] and 0.933 versus 0.829 [*P = *0.003] for CUIMC and non-CUIMC genomes, respectively) (Data Set S1, sheet 10).

### Understanding determinants of PR through machine learning.

Being able to interpret how ML algorithms arrive at their conclusions remains a central challenge to applying ML approaches in clinical decision-making, including AST genotype-phenotype prediction (18). We therefore wanted to interpret ML models to assess whether they are biologically plausible and incorporate known PR determinants, as well as to use them to identify potential novel determinants of PR. We were able to extract a ranking of genes according to their relative importance to the model (model-specific feature importance) for logistic regression, random forests, and GBTC but not for SVC due to the nature of the model.

We focused on the GBTC model trained on all genomes by using a reference-based approach due to the diversity of the genomes and its high performance. The genes ranked highest in feature importance, and their quantitative feature importance metrics are listed in Table 3. Feature importance results for data sets using CUIMC and non-CUIMC genomes only are included in Data Set S1, sheet 11. With no prior programming, the model identified all canonical PR genes except *crrA* and ranked *mgrB*, *phoQ*, *pmrA*, and *pmrB* as the four genes with the highest feature importance.

**TABLE 3:** Genes ranked by relative feature importance in ML model for PR prediction that incorporated all genomes and used GBTC^*a*^

Annotation	Quantitative feature importance metric	Full name	Function and comments	Reference(s)
*mgrB*	0.317		Known determinant	
*phoQ*	0.0454		Known determinant	
*pmrA*	0.0343		Known determinant	
*pmrB*	0.0325		Known determinant	
*lpdA*	0.0284	Dihydrolipoyl dehydrogenase	Respiratory chain enzyme; implicated in response to polymyxin B	72
*ahpF*	0.0250	Alkyl hydroperoxide reductase subunit F	Outer membrane protein conferring hydrogen peroxide resistance and implicated in stress response	73
*grxD*	0.0195	Grx4 family monothiol glutaredoxin	Iron regulation	74
*envZ*	0.0185		Sensing of osmotic signals, regulation of biofilm formation and capsule production; implicated in response to polymyxin B	72, 75, 76
*pstB*	0.0183	Phosphate import ATP- binding protein PstB	Capture and transport of periplasmic phosphate into cell	77
*crrB*	0.0160		Known determinant	
*dmlR_5*	0.0147		Unknown	
*phoP*	0.0143		Known determinant	
*arnA*	0.0136	UDP 4-deoxy-4- formamido-l-arabinose transferase	Part of *arn* operon that attaches arabinose to lipid A to confer polymyxin resistance	33
*pepN*	0.0136	Aminopeptidase N	Cell wall protein, possible target of neutrophil elastase	78
*pqiB_2*	0.0133	Paraquat-inducible protein B	Involved in transport pathways that contribute to membrane integrity	79
KP0228_00228	0.0113	H239_3063	Encodes putative RND-type efflux pump and newly discovered determinant of PR	32
*gyrB*	0.0104	DNA topoisomerase (ATP- hydrolyzing) subunit B	Implicated in fluoroquinolone resistance	80
KP0228_00219	0.00954		Unknown	80
*cytR_1*	0.00902		Unknown	
*pgpB*	0.00897	Phosphatidylglycero- phosphatase B	Involved in generating phospholipid for cell membrane	81, 82

a ML, machine learning; PR, polymyxin resistance; GBTC, gradient-boosted trees classifier.

In addition to identifying these known determinants, we assessed other highly ranked genes as candidate novel determinants of PR by conducting a literature search (Table 3). Several genes have been noted to have potential roles in PR previously, including H239_3063, which encodes a newly identified putative RND-type efflux pump (32), and *arnA*, which is part of the *arn* operon that attaches arabinose to lipid A to confer PR (33). Many of the other genes encode outer membrane proteins or have functions that may affect the cell membrane and thus are possibly involved in interactions with polymyxins, including *lpdA*, *ahpF*, *envZ*, *pstB*, *pepN*, and *pgpB*.

## DISCUSSION

Our study provides a proof of principle demonstrating the utility of using ML for prediction of phenotypic PR from genomic data and raises important methodological issues that have implications for the use of ML for AST genotype-phenotype prediction more generally. We focused efforts on PR due to the clinical need posed by resistance to this class of last-line antimicrobials, the difficulties associated with performing phenotypic AST for polymyxins, and the complexity of underlying resistance mechanisms that may be uniquely suited to ML approaches. We noted high model performance across a range of metrics by leveraging a large collection of PR isolates from our institution and identifying publicly available genomes with BMD phenotypic susceptibility data. This performance was achieved through the use of reference-based input data sets, the use of GWAS as a filtering procedure, and the addition of polymyxin exposure as a clinical variable. We also were able to interpret ML models to confirm biological plausibility and identify additional potential genetic determinants of PR as candidates for functional testing.

Representation and selection of genomic features, a process termed “feature engineering” in the ML literature, was central to the success of resistance prediction and could affect performance both positively and negatively. First, our reference-based approach had high performance (AUROC, 0.885 to 0.933) and outperformed the rule-based approach that we used as a benchmark. Despite PR being more biologically complex than other forms of antimicrobial resistance in K. pneumoniae, it was encouraging that our results were similar to those obtained for other antimicrobials with less complex mechanisms of resistance (e.g., carbapenem resistance) tested with ML approaches (14, 15, 34–36). The performance of this approach falls below the FDA cutoffs for AST tests (37). However, given the problematic nature of phenotypic polymyxin susceptibility testing, it may help to identify a subset of genomes with a high likelihood of PR for confirmatory phenotypic testing.

In the setting of this high performance with our baseline approach, it was difficult to demonstrate a statistically significant improvement using additional feature engineering. With this in mind, we noted a moderate rise across nearly all metrics using GWAS filtering, when both reference-based and reference-free approaches were used. The role of GWAS filtering for improving performance in other AST genotype-phenotype prediction settings remains to be determined. AST genotype-phenotype data sets typically have a small number of genomes with a large number of genomic features; therefore, GWAS filtering provides a biologically consistent way of selecting genomic features. This approach has not been extensively used in AST genotype-phenotype prediction, although it has been used to predict resistance in Streptococcus pneumoniae (38). In addition, it has been used to predict other bacterial phenotypes (39, 40) and in other nonmicrobiological studies (41–43).

In contrast to filtering unnecessary genomic features, we noted that adding a single pertinent clinical feature in the form of polymyxin exposure data may increase performance by an amount similar to that of the more complex GWAS filtering approach, although the same caveats regarding a lack of statistical significance apply. MacFadden et al. used prior antimicrobial exposure data with genotypic data for ML prediction of phenotype of Escherichia coli susceptibility to three different antimicrobial classes and similarly noted an increase in performance over that of using genotypic data alone (16). We focused on prior polymyxin exposure, as it is a known epidemiological risk factor and also as a proof of principle, because drug administration is represented by highly structured variables in electronic medical records. In a recent retrospective cohort study of PR isolates at a single institution from 2011 to 2016, neurologic disease, residence in a skilled nursing facility prior to admission, receipt of carbapenems in the last 90 days, prior infection with a carbapenem-resistant organism, and receipt of ventilatory support were risk factors for PR in a multivariate model (44). Potentially, any of these clinical variables might be added to the model when available. We envision that the widespread adoption of electronic medical records will allow more extensive and automated integration of such clinical and genotypic data, thus enabling more accurate prediction both of AST phenotypes and other outcomes.

Certain forms of representation of genetic data may also have a negative impact on performance. While the use of reference-free approaches for feature representation incorporating k-mers has been the focus of much recent work (14, 15, 31, 34, 35, 45–48), we noted that reference-based approaches using variant calling and IS detection had significantly better performance overall. This may reflect the biological basis for PR, with IS elements playing an important role, particularly in association with *mgrB* (6, 8, 12, 49). k-mer-based approaches may have difficulty accurately detecting this, due to the diversity of IS elements in *K. pneumoniae* genomes, as well as the possibility that they insert in different sites in individual genes or in regulatory regions upstream (50). Additionally, reference-free approaches take the accessory genome into account. This offers putative benefits in terms of detecting various mobile genetic elements and making it easier to use this approach across diverse genomes. However, these benefits may be offset by the additional “noise” in the case of organisms with large accessory genomes, such as K. pneumoniae. Hicks et al. also found that ML approaches had worse performance when attempting to predict ciprofloxacin resistance in organisms with larger pan-genomes (K. pneumoniae and Acinetobacter) than in Neisseria gonorrhoeae (15). While k-mer-based approaches may work well when the mechanism largely depends on a single or a few resistance determinants (e.g., beta-lactamases), our findings suggest that a more curated approach may be needed for more complex forms of resistance such as PR.

Beyond these biological issues, use of reference-free input data also has important computational consequences. First, it increased the number of features by orders of magnitude in comparison with using reference-based approaches that use genes as inputs. In order to attenuate that effect, we incorporated feature selection hyperparameters in our pipeline, but given the limited number of genomes for analysis, the increased dimensionality nevertheless likely negatively affected algorithm performance. The large number of features also requires more computationally intensive analyses, leading to much longer run times and raising questions about how plausible it would be to run these analyses as part of a clinical workflow. In order to address some of these computational issues, Drouin et al. adapted the set covering machine algorithm and implemented it in the KOVER package (31). When we used our data with KOVER, the run time was much lower than that of our pipeline, but model performance was similarly low.

In contrast to our findings regarding the importance of feature engineering, surprisingly neither the data used to train ML algorithms (in the form of input genomes) nor the choice of specific ML algorithm significantly impacted model performance. With regard to the input genomes used for training, this was in contrast to the sampling bias noted by Hicks et al. (15) and was somewhat surprising, as the CUIMC isolates had a high degree of clonal relatedness and rates of PR varied depending on the origin data set selected (Fig. 1). Similarly, Moradigaravand et al. hypothesized that ensemble algorithms such as GBTC or random forests may be better suited to AST genotype-phenotype prediction (17), but we did not observe these differences. Our different findings may be explained by the fact that these studies focused on different organisms (N. gonorrhoeae and E. coli, respectively) and different antimicrobials. Our approach of representing genomes as binary variants in a reference’s coding regions, rather than focusing on individual variants, may also have led to more robust results. This further underlines prior concerns about attempting to use a “one-size-fits-all” approach, given the diverse biological underpinnings of antimicrobial resistance (15).

The choice of performance metric to be used in AST genotype-phenotype prediction also remains an open question, with no consensus between prior studies (13). Indeed, the choice may be dictated by the expected prevalence of the phenotype of interest and the intended use of the ML model. Our intention was to create a screening method where isolates could be identified for confirmatory phenotypic testing, and hence we chose AUROC, as it is an aggregative metric that looks at the balance between the true-positive and false-positive rates. It has been used in other AST genotype-phenotype studies (16, 34, 45–47, 51–53) and is a metric commonly used to assess clinical test performance. However, it may not be the optimal metric if there is significant imbalance in the data set (54), leading Hicks et al. to advocate for use of multiple metrics (15). We therefore reported multiple metrics, including precision (equivalent to positive predictive value), which we felt was important for our use case, as it helps to assess how many isolates identified as resistant by the ML algorithm are phenotypically resistant. Using GWAS filtering, we achieved a precision of ∼90%.

Our best-performing ML models were also interpretable, thus allowing us to confirm that predictions were biologically plausible. The models correctly identified canonical PR genes, with the exception of *crrA*, which had a low prevalence of variants across all data sets. This is highly encouraging, given that this comprises six genes, with variants in multiple canonical genes often occurring in PR isolates (10, 12). In addition, they also confirmed genes that had been associated with PR in more limited settings, including H239_3063 and *arnA.* Finally, several potential novel determinants were identified through the use of ML, with multiple genes involved in stress responses or maintenance of the cell membrane. Although beyond the scope of this study, these determinants now require functional testing to prove causality, and it is possible that exposure to other antimicrobials may have contributed to the observed genetic changes. This is demonstrated by the fact that the ML model incorporating all genomes identified *gyrB*, which has been associated with fluoroquinolone resistance.

Our study had several limitations, particularly related to the use of a reference-based approach. First, the representation of genomes as binary variants in a reference’s coding regions is an intentional simplification. An obvious problem is that all variants are treated as equal, whereas our previous work has shown that some variants may not have a functional impact, particularly in *phoQ* (12). However, this appears to be a reasonable trade-off, given the limited number of genomes available for analysis. Alternative approaches may include using individual alleles or a weighted score that takes into account likelihood of functional impact based on amino acid changes, rather than a simple binary representation. A second limitation is our use of a single reference chromosome. This limits generalizability to CG258 genomes, which in future work may be overcome through attempting to define a K. pneumoniae core genome for analysis. Holt et al. attempted to do this and noted 1,888 “common” genes that were present in ≥95% of 328 K. pneumoniae genomes (55). However, each genome carries thousands of additional accessory genes, likely making it difficult to apply this approach to K. pneumoniae as an entire species. The use of a reference-based approach also entails that nonchromosomal regions are not incorporated into models. While plasmid-mediated PR is rare in K. pneumoniae (and was checked for specifically in our collection), *mcr* genes or another plasmid-based determinant would not be detected by our reference-based approach. An additional consideration is the impact of population structure, particularly in single-institution data sets which may contain closely related genomes. While this has been well described as a potential confounder in bacterial GWAS (56), it has not been addressed in AST genotype-phenotype prediction but may play a similarly confounding role (15).

In summary, our study demonstrated that ML methods can achieve high performance for prediction of phenotypic PR in K. pneumoniae CG258, even in the face of PR being a remarkably polygenic trait. In contrast to other recent work on AST genotype-phenotype prediction, we noted best performance through the use of a reference-based and curated input data set, which may reflect the underlying biological complexity of PR and be applicable to other complex forms of antimicrobial resistance. We noted that incorporating GWAS as a filtering procedure and addition of clinical data on antimicrobial exposure may improve performance, but these findings need confirmation in other settings. The increasing availability of genomic data makes AST genotype-phenotype prediction an important priority, and use of ML will need to be tailored to specific organisms and antimicrobial agents.

## MATERIALS AND METHODS

### Genome selection.

The study was reviewed and approved by the CUIMC Institutional Review Board. We included K. pneumoniae CG258 isolates, comprising K. pneumoniae ST258 and multilocus sequence type single-locus variants (e.g., ST11 and ST512). CUIMC isolates were selected as previously described and comprised K. pneumoniae CG258 isolates spanning 2011 to 2018 (12). All CUIMC isolates had MIC determination with BMD according to CLSI guidelines (57). We performed WGS on all included CUIMC isolates as described previously (12, 58–60). In total, we included 313 K. pneumoniae CG258 genomes. We then identified publicly available K. pneumoniae genomes by searching PubMed with the terms “colistin resistance” and “polymyxin resistance.” We also searched the CDC/FDA Antibiotic Resistance Isolate Bank and PATRIC databases (20, 25). When possible, we obtained genome raw sequence data from NCBI in preference to draft assemblies. Genomes with non-BMD phenotypic susceptibility testing data were excluded due to concerns about accuracy (2). See Fig. 1 and Data Set S1, sheet 1, for all accession numbers, source data sets, phenotypic susceptibility, and specific multilocus sequence types. We included 306 publicly available K. pneumoniae CG258 genomes. For each analysis, we tested all 619 genomes and then subsets of 313 CUIMC genomes and 306 non-CUIMC genomes. We constructed and visualized a phylogeny of all included isolates, as described previously (12).

MIC ranges varied according to susceptibility testing platform (e.g., SensiTitre versus manual methods). We therefore used a qualitative definition of polymyxin susceptibility, with isolates considered PR if the colistin or polymyxin B MIC was >2 mg/liter (57, 61). We constructed draft *de novo* assemblies of all genomes with raw sequence data using the Shovill wrapper for SPAdes (62) and then used Kleborate for multilocus sequence type (MLST) and resistance determinant (including *mcr* genes) detection (63, 64). No *mcr* genes were detected.

### Data set preparation.

A reference-based approach and a reference-free approach were used to create input matrices for ML algorithms. The outcome of interest (“label” in ML literature) for both was polymyxin susceptibility as a binary outcome (susceptible/resistant). For the reference-based approach, we had selected a representative polymyxin-susceptible CG258 isolate and created a *de novo* hybrid assembly (12, 65) that was then used to create a profile of each genome through a combination of variant calling and IS detection, as described previously (12). In brief, variant calling was performed using Snippy 3.2 and running Snippy in the “– contig” mode if only an assembly was available (66). ISseeker was used to identify sites with IS elements present (50). In order to increase the sensitivity of detection for key genes, we also used a BLAST database of PR canonical genes to identify if IS disruption or large-scale deletions of these genes may have occurred.

These analyses allowed us to identify CG258 isolates containing SNVs/IS in coding regions relative to the reference genome. Intergenic regions, synonymous SNVs in coding regions, and SNVs in known mobile genetic elements and repeat regions were not considered. In the data set including all genomes, we identified 14,783 variants in 3,653 genes; however, 7,149 of 14,783 (48.3%) variants occurred in single isolates. Therefore, we incorporated individual genes rather than variants, scoring each of these alleles in each isolate as 1 if it differed from the reference and 0 if it did not. These allele scores were used to create a binary matrix, with isolates as rows and coding regions in the reference as columns. We also integrated clinical data about polymyxin exposure from our prior study for CUIMC isolates and created a binary variable that indicated polymyxin exposure at any time in that patient prior to culture of the isolate (12).

We constructed a reference-free input matrix based on k-mer profiles generated with the DSK k-mer counting software with *k* equal to 31, a length commonly used in bioinformatics analyses (14, 15, 30, 31, 34, 67, 68) that may maximize association power in bacterial genomics studies (15, 69, 70). A k-mer presence/absence binary matrix was then created and used as an input for ML analyses.

### Rule-based prediction of polymyxin resistance.

We used rule-based prediction as a baseline for comparison with ML approaches. The SNV/IS detection results described for reference-based data sets described above were used to find variants in any of the canonical PR genes (*crrAB*, *mgrB*, *pmrAB*, *phoPQ*) that have been established as key contributors to PR (5–11). Isolates were then classified as PR if there was a variant present in any of these genes.

### Use of genome-wide association study for feature filtering.

As an additional feature engineering for the reference-based approach, we filtered features using *P* values from a GWAS. The reference-based binary matrix was used as input in treeWAS, an R package for bacterial GWAS (27). treeWAS was run in the “simultaneous” mode to account for population structure. The resulting *P* values were used to rank and select genomic features under a specific *P* value threshold (see below), with the rest discarded.

### Machine learning analyses.

We created a bespoke pipeline using scikit-learn (26), which performed additional feature selection and hyperparameter tuning (code available on GitHub at https://github.com/crowegian/AMR_ML). Hyperparameters were tuned with 10-fold cross-validation (CV) using AUROC as the performance metric. Reference-based data sets without GWAS filtering and reference-free data sets used a linear support vector classifier to extract important features for feature selection, also with 10-fold CV. As GWAS *P* values were generated using labeled data, in order to prevent data leakage for reference-based data sets with GWAS filtering, we did not use cross validation. Instead, we used a 75%-25% training-validation split to tune hyperparameters where the GWAS values and models were trained using the 75% split training data. All data sets made use of a linear support vector classifier to filter features. Data sets with GWAS *P* values also used *P* values to filter features by making them a hyperparameter tuned from a minimum allowable *P* value of 0 to the 75th percentile of all *P* values in steps of 0.01. For data sets with GWAS filtering, features chosen by either step were all selected. Hyperparameters were tuned for each data set across the feature selection step and for each model configuration.

We tested four different ML models: logistic regression, random forests, SVC, and GBTC. The performance metrics used were AUROC, balanced accuracy, accuracy, precision, recall, and F1 score. Best-performing models were chosen based on AUROC, as this is an aggregative metric and has been used in prior studies of AST genotype-phenotype prediction (16, 34, 45–47, 51–53).

We then also compared the results of our ML pipeline to the ML implementation in the KOVER package that utilizes the set covering machine and classification and regression trees algorithms (14, 31). For the KOVER implementation, the best conjunctive and/or disjunctive model was selected using 10-fold cross-validation, testing the suggested broad range of values for the trade-off hyperparameter of 0.1, 0.178, 0.316, 0.562, 1.0, 1.778, 3.162, 5.623, 10.0, 144, and 999,999.0 to determine the optimal rule scoring function with default parameters. The pROC R package was used to calculate AUROC for the predictions made by the rule-based algorithm and KOVER package (71).

### Statistical testing.

Cross-validation results were used to generate means and 95% confidence intervals for all performance metrics. As models trained using the 75%-25% split with GWAS *P* values do not have sensical confidence intervals, bootstrapping was performed. Ten bootstrapped training and validation sets were created by pooling the split data and repeatedly bootstrapping (sampling *n* observations with replacement) a training data set from the original data and then using the rest of the data as a validation set. The best model and its hyperparameters for each model class (logistic regression, SVC, etc.) were chosen based on the original 75%-25% split performance and then retrained and evaluated on each of the bootstrapped training-validation pairs, and metrics were collected. Model performance was evaluated by comparing mean performance metrics using two-tailed *t* tests or Mann-Whitney U tests, as appropriate. Categorical variables were compared using χ^2^ or Fisher’s exact tests, as appropriate. ANOVA was used for comparison of means between multiple groups. Statistical analyses were performed in R (v3.4.0).

### Data availability.

All CUIMC genomes have been deposited in the NCBI Sequence Read Archive under NCBI BioProject accession numbers PRJNA557275 and PRJNA445400. For individual accession numbers, please see Data Set S1, sheet 1, in the supplemental material.

## References

[1] Poirel L, Jayol A, Nordmann P 2017 Polymyxins: antibacterial activity, susceptibility testing, and resistance mechanisms encoded by plasmids or chromosomes. Clin Microbiol Rev 30:557–596. doi:10.1128/CMR.00064-16. 28275006 PMC5355641

[2] Hindler JA, Humphries RM 2013 Colistin MIC variability by method for contemporary clinical isolates of multidrug-resistant Gram-negative bacilli. J Clin Microbiol 51:1678–1684. doi:10.1128/JCM.03385-12. 23486719 PMC3716094

[3] Humphries RM 2015 Susceptibility testing of the polymyxins: where are we now? Pharmacotherapy 35:22–27. doi:10.1002/phar.1505. 25329490

[4] Humphries RM, Green DA, Schuetz AN, Bergman Y, Lewis S, Yee R, Stump S, Lopez M, Macesic N, Uhlemann AC, Kohner P, Cole N, Simner PJ 2019 Multi-center evaluation of colistin broth disk elution and colistin agar test: a report from the Clinical and Laboratory Standards Institute. J Clin Microbiol 57doi:10.1128/JCM.01269–19. doi:10.1128/JCM.01269-19. PMC6813006 31511331

[5] Aires CA, Pereira PS, Asensi MD, Carvalho-Assef AP 2016 mgrB mutations mediating polymyxin B resistance in Klebsiella pneumoniae isolates from rectal surveillance swabs in Brazil. Antimicrob Agents Chemother 60:6969–6972. doi:10.1128/AAC.01456-16. 27620478 PMC5075120

[6] Berglund B, Hoang NTB, Tarnberg M, Le NK, Svartstrom O, Khu DTK, Nilsson M, Le HT, Welander J, Olson L, Larsson M, Nilsson LE, Hanberger H 2018 Insertion sequence transpositions and point mutations in mgrB causing colistin resistance in a clinical strain of carbapenem-resistant Klebsiella pneumoniae from Vietnam. Int J Antimicrob Agents 51:789–793. doi:10.1016/j.ijantimicag.2017.11.012. 29180281

[7] Cannatelli A, Di Pilato V, Giani T, Arena F, Ambretti S, Gaibani P, D'Andrea MM, Rossolini GM 2014 In vivo evolution to colistin resistance by PmrB sensor kinase mutation in KPC-producing Klebsiella pneumoniae is associated with low-dosage colistin treatment. Antimicrob Agents Chemother 58:4399–4403. doi:10.1128/AAC.02555-14. 24841267 PMC4136067

[8] Cannatelli A, COLGRIT Study Group, Giani T, D’Andrea MM, Di Pilato V, Arena F, Conte V, Tryfinopoulou K, Vatopoulos A, Rossolini GM, Group CS 2014 MgrB inactivation is a common mechanism of colistin resistance in KPC-producing Klebsiella pneumoniae of clinical origin. Antimicrob Agents Chemother 58:5696–5703. doi:10.1128/AAC.03110-14. 25022583 PMC4187966

[9] Cheng YH, Lin TL, Pan YJ, Wang YP, Lin YT, Wang JT 2015 Colistin resistance mechanisms in Klebsiella pneumoniae strains from Taiwan. Antimicrob Agents Chemother 59:2909–2913. doi:10.1128/AAC.04763-14. 25691646 PMC4394772

[10] Pitt ME, Elliott AG, Cao MD, Ganesamoorthy D, Karaiskos I, Giamarellou H, Abboud CS, Blaskovich MAT, Cooper MA, Coin L 2018 Multifactorial chromosomal variants regulate polymyxin resistance in extensively drug-resistant Klebsiella pneumoniae. Microb Genom 4:e000158. doi:10.1099/mgen.0.000158. PMC5885010 29431605

[11] Wright MS, Suzuki Y, Jones MB, Marshall SH, Rudin SD, van Duin D, Kaye K, Jacobs MR, Bonomo RA, Adams MD 2015 Genomic and transcriptomic analyses of colistin-resistant clinical isolates of Klebsiella pneumoniae reveal multiple pathways of resistance. Antimicrob Agents Chemother 59:536–543. doi:10.1128/AAC.04037-14. 25385117 PMC4291396

[12] Macesic N, Nelson B, McConville TH, Giddins MJ, Green DA, Stump S, Gomez-Simmonds A, Annavajhala MK, Uhlemann AC 2019 Emergence of polymyxin resistance in clinical Klebsiella pneumoniae through diverse genetic adaptations: a genomic, retrospective cohort study. Clin Infect Dis doi:10.1093/cid/ciz623. PMC7201408 31513705

[13] Su M, Satola SW, Read TD 2018 Genome-based prediction of bacterial antibiotic resistance. J Clin Microbiol 57:2018–2015. doi:10.1128/JCM.01405-18. PMC6425178 30381421

[14] Drouin A, Letarte G, Raymond F, Marchand M, Corbeil J, Laviolette F 2019 Interpretable genotype-to-phenotype classifiers with performance guarantees. Sci Rep 9:4071. doi:10.1038/s41598-019-40561-2. 30858411 PMC6411721

[15] Hicks AL, Wheeler N, Sanchez-Buso L, Rakeman JL, Harris SR, Grad YH 2019 Evaluation of parameters affecting performance and reliability of machine learning-based antibiotic susceptibility testing from whole genome sequencing data. PLoS Comput Biol 15:e1007349. doi:10.1371/journal.pcbi.1007349. 31479500 PMC6743791

[16] MacFadden DR, Melano RG, Coburn B, Tijet N, Hanage WP, Daneman N 2019 Comparing patient risk factor-, sequence type-, and resistance locus identification-based approaches for predicting antibiotic resistance in Escherichia coli bloodstream infections. J Clin Microbiol 57:e01780-18. doi:10.1128/JCM.01780-18. 30894438 PMC6535602

[17] Moradigaravand D, Palm M, Farewell A, Mustonen V, Warringer J, Parts L 2018 Prediction of antibiotic resistance in Escherichia coli from large-scale pan-genome data. PLoS Comput Biol 14:e1006258-17. doi:10.1371/journal.pcbi.1006258. 30550564 PMC6310291

[18] Macesic N, Polubriaginof F, Tatonetti NP 2017 Machine learning: novel bioinformatics approaches for combating antimicrobial resistance. Curr Opin Infect Dis 30:511–517. doi:10.1097/QCO.0000000000000406. 28914640

[19] Pitout JDD, Nordmann P, Poirel L 2015 Carbapenemase-producing Klebsiella pneumoniae, a key pathogen set for global nosocomial dominance. Antimicrob Agents Chemother 59:5873–5884. doi:10.1128/AAC.01019-15. 26169401 PMC4576115

[20] Centers for Disease Control and Prevention. 2019 CDC & FDA Antibiotic Resistance (AR) Isolate Bank. https://www.cdc.gov/drugresistance/resistance-bank/index.html. Accessed 15 August 2019.

[21] Deleo FR, Chen L, Porcella SF, Martens CA, Kobayashi SD, Porter AR, Chavda KD, Jacobs MR, Mathema B, Olsen RJ, Bonomo RA, Musser JM, Kreiswirth BN 2014 Molecular dissection of the evolution of carbapenem-resistant multilocus sequence type 258 Klebsiella pneumoniae. Proc Natl Acad Sci U S A 111:4988–4993. doi:10.1073/pnas.1321364111. 24639510 PMC3977278

[22] Rimoldi SG, Gentile B, Pagani C, Di Gregorio A, Anselmo A, Palozzi AM, Fortunato A, Pittiglio V, Ridolfo AL, Gismondo MR, Rizzardini G, Lista F 2017 Whole genome sequencing for the molecular characterization of carbapenem-resistant Klebsiella pneumoniae strains isolated at the Italian ASST Fatebenefratelli Sacco Hospital, 2012–2014. BMC Infect Dis 17:666. doi:10.1186/s12879-017-2760-7. 29017452 PMC5634883

[23] Stoesser N, Giess A, Batty EM, Sheppard AE, Walker AS, Wilson DJ, Didelot X, Bashir A, Sebra R, Kasarskis A, Sthapit B, Shakya M, Kelly D, Pollard AJ, Peto TE, Crook DW, Donnelly P, Thorson S, Amatya P, Joshi S 2014 Genome sequencing of an extended series of NDM-producing Klebsiella pneumoniae isolates from neonatal infections in a Nepali hospital characterizes the extent of community- versus hospital-associated transmission in an endemic setting. Antimicrob Agents Chemother 58:7347–7357. doi:10.1128/AAC.03900-14. 25267672 PMC4249533

[24] van Dorp L, Wang Q, Shaw LP, Acman M, Brynildsrud OB, Eldholm V, Wang R, Gao H, Yin Y, Chen H, Ding C, Farrer RA, Didelot X, Balloux F, Wang H 2019 Rapid phenotypic evolution in multidrug-resistant Klebsiella pneumoniae hospital outbreak strains. Microb Genom 5:e000263. doi:10.1099/mgen.0.000263. PMC6521586 30939107

[25] Wattam AR, Davis JJ, Assaf R, Boisvert S, Brettin T, Bun C, Conrad N, Dietrich EM, Disz T, Gabbard JL, Gerdes S, Henry CS, Kenyon RW, Machi D, Mao C, Nordberg EK, Olsen GJ, Murphy-Olson DE, Olson R, Overbeek R, Parrello B, Pusch GD, Shukla M, Vonstein V, Warren A, Xia F, Yoo H, Stevens RL 2017 Improvements to PATRIC, the all-bacterial Bioinformatics Database and Analysis Resource Center. Nucleic Acids Res 45:D535–D542. doi:10.1093/nar/gkw1017. 27899627 PMC5210524

[26] Pedregosa F, Varoquaux G, Gramfort A, Michel V, Thirion B, Grisel O, Blondel M, Müller A, Nothman J, Louppe G, Prettenhofer P, Weiss R, Dubourg V, Vanderplas J, Passos A, Cournapeau D, Brucher M, Perrot M, Duchesnay É 2011 Scikit-learn: machine learning in Python. J Machine Learning Res 12:2825–2830.

[27] Collins C, Didelot X 2018 A phylogenetic method to perform genome-wide association studies in microbes that accounts for population structure and recombination. PLoS Comput Biol 14:e1005958. doi:10.1371/journal.pcbi.1005958. 29401456 PMC5814097

[28] Rajkomar A, Dean J, Kohane I 2019 Machine learning in medicine. N Engl J Med 380:1347–1358. doi:10.1056/NEJMra1814259. 30943338

[29] Giacobbe DR, ISGRI-SITA (Italian Study Group on Resistant Infections of the Società Italiana Terapia Antinfettiva), Del Bono V, Trecarichi EM, De Rosa FG, Giannella M, Bassetti M, Bartoloni A, Losito AR, Corcione S, Bartoletti M, Mantengoli E, Saffioti C, Pagani N, Tedeschi S, Spanu T, Rossolini GM, Marchese A, Ambretti S, Cauda R, Viale P, Viscoli C, Tumbarello M, Isgri S 2015 Risk factors for bloodstream infections due to colistin-resistant KPC-producing Klebsiella pneumoniae: results from a multicenter case-control-control study. Clin Microbiol Infect 21:1106.E1–1108.E8. doi:10.1016/j.cmi.2015.08.001. 26278669

[30] Rizk G, Lavenier D, Chikhi R 2013 DSK: k-mer counting with very low memory usage. Bioinformatics 29:652–653. doi:10.1093/bioinformatics/btt020. 23325618

[31] Drouin A, Giguere S, Deraspe M, Marchand M, Tyers M, Loo VG, Bourgault AM, Laviolette F, Corbeil J 2016 Predictive computational phenotyping and biomarker discovery using reference-free genome comparisons. BMC Genomics 17:754. doi:10.1186/s12864-016-2889-6. 27671088 PMC5037627

[32] Cheng YH, Lin TL, Lin YT, Wang JT 2018 A putative RND-type efflux pump, H239_3064, contributes to colistin resistance through CrrB in Klebsiella pneumoniae. J Antimicrob Chemother 73:1509–1516. doi:10.1093/jac/dky054. 29506266 PMC5961088

[33] Gatzeva-Topalova PZ, May AP, Sousa MC 2005 Structure and mechanism of ArnA: conformational change implies ordered dehydrogenase mechanism in key enzyme for polymyxin resistance. Structure 13:929–942. doi:10.1016/j.str.2005.03.018. 15939024 PMC2997725

[34] Long SW, Olsen RJ, Eagar TN, Beres SB, Zhao P, Davis JJ, Brettin T, Xia F, Musser JM 2017 Population genomic analysis of 1,777 extended-spectrum beta-lactamase-producing Klebsiella pneumoniae isolates, Houston, Texas: unexpected abundance of clonal group 307. mBio 8:e00489-17. doi:10.1128/mBio.00489-17. 28512093 PMC5433097

[35] Nguyen M, Brettin T, Long SW, Musser JM, Olsen RJ, Olson R, Shukla M, Stevens RL, Xia F, Yoo H, Davis JJ 2018 Developing an in silico minimum inhibitory concentration panel test for Klebsiella pneumoniae. Sci Rep 8:421. doi:10.1038/s41598-017-18972-w. 29323230 PMC5765115

[36] Pesesky MW, Hussain T, Wallace M, Patel S, Andleeb S, Burnham C-A, Dantas G 2016 Evaluation of machine learning and rules-based approaches for predicting antimicrobial resistance profiles in Gram-negative bacilli from whole genome sequence data. Front Microbiol 7:1887–1817. doi:10.3389/fmicb.2016.01887. 27965630 PMC5124574

[37] Humphries RM, Ambler J, Mitchell SL, Castanheira M, Dingle T, Hindler JA, Koeth L, Sei K, Hardy D, Zimmer B, Butler-Wu S, Dien Bard J, Brasso B, Shawar R, Dingle T, Humphries R, Sei K, Koeth L, Standardization Working Group of the Subcommittee on Antimicrobial Susceptibility Testing. 2018 CLSI Methods Development and Standardization Working Group best practices for evaluation of antimicrobial susceptibility tests. J Clin Microbiol 56:e01934-17. doi:10.1128/JCM.01934-17. 29367292 PMC5869819

[38] Mobegi FM, Cremers AJ, de Jonge MI, Bentley SD, van Hijum SA, Zomer A 2017 Deciphering the distance to antibiotic resistance for the pneumococcus using genome sequencing data. Sci Rep 7:42808. doi:10.1038/srep42808. 28205635 PMC5311915

[39] Meric G, Mageiros L, Pensar J, Laabei M, Yahara K, Pascoe B, Kittiwan N, Tadee P, Post V, Lamble S, Bowden R, Bray JE, Morgenstern M, Jolley KA, Maiden MCJ, Feil EJ, Didelot X, Miragaia M, de Lencastre H, Moriarty TF, Rohde H, Massey R, Mack D, Corander J, Sheppard SK 2018 Disease-associated genotypes of the commensal skin bacterium Staphylococcus epidermidis. Nat Commun 9:5034. doi:10.1038/s41467-018-07368-7. 30487573 PMC6261936

[40] Recker M, Laabei M, Toleman MS, Reuter S, Saunderson RB, Blane B, Török ME, Ouadi K, Stevens E, Yokoyama M, Steventon J, Thompson L, Milne G, Bayliss S, Bacon L, Peacock SJ, Massey RC 2017 Clonal differences in Staphylococcus aureus bacteraemia-associated mortality. Nat Microbiol 2:1381–1388. doi:10.1038/s41564-017-0001-x. 28785103

[41] Okser S, Pahikkala T, Aittokallio T 2013 Genetic variants and their interactions in disease risk prediction—machine learning and network perspectives. BioData Min 6:5. doi:10.1186/1756-0381-6-5. 23448398 PMC3606427

[42] Wei Z, Wang K, Qu H-Q, Zhang H, Bradfield J, Kim C, Frackleton E, Hou C, Glessner JT, Chiavacci R, Stanley C, Monos D, Grant SFA, Polychronakos C, Hakonarson H 2009 From disease association to risk assessment: an optimistic view from genome-wide association studies on type 1 diabetes. PLoS Genet 5:e1000678-11. doi:10.1371/journal.pgen.1000678. 19816555 PMC2748686

[43] Wei Z, Wang W, Bradfield J, Li J, Cardinale C, Frackelton E, Kim C, Mentch F, Van Steen K, Visscher PM, Baldassano RN, Hakonarson H, International IBD Genetics Consortium. 2013 Large sample size, wide variant spectrum, and advanced machine-learning technique boost risk prediction for inflammatory bowel disease. Am J Hum Genet 92:1008–1012. doi:10.1016/j.ajhg.2013.05.002. 23731541 PMC3675261

[44] Richter SE, Miller L, Uslan DZ, Bell D, Watson K, Humphries R, McKinnell JA 2018 Risk factors for colistin resistance among Gram-negative rods and Klebsiella pneumoniae isolates. J Clin Microbiol 56:e00149-18. doi:10.1128/JCM.00149-18. 29976595 PMC6113453

[45] Davis JJ, Boisvert S, Brettin T, Kenyon RW, Mao C, Olson R, Overbeek R, Santerre J, Shukla M, Wattam AR, Will R, Xia F, Stevens R 2016 Antimicrobial resistance prediction in PATRIC and RAST. Sci Rep 6:27930. doi:10.1038/srep27930. 27297683 PMC4906388

[46] Mahé P, Tournoud M 2018 Predicting bacterial resistance from whole-genome sequences using k-mers and stability selection. BMC Bioinformatics 19:383. doi:10.1186/s12859-018-2403-z. 30332990 PMC6192184

[47] Mahé P, El Azami M, Barlas P, Tournoud M 2019 A large scale evaluation of TBProfiler and Mykrobe for antibiotic resistance prediction in Mycobacterium tuberculosis. PeerJ 7:e6857-21. doi:10.7717/peerj.6857. 31106066 PMC6500375

[48] Nguyen M, Long SW, McDermott PF, Olsen RJ, Olson R, Stevens RL, Tyson GH, Zhao S, Davis JJ 2018 Using machine learning to predict antimicrobial MICs and associated genomic features for nontyphoidal Salmonella. J Clin Microbiol 57:495–415. doi:10.1128/JCM.01260-18. PMC6355527 30333126

[49] Arena F, Henrici De Angelis L, Cannatelli A, Di Pilato V, Amorese M, D'Andrea MM, Giani T, Rossolini GM 2016 Colistin resistance caused by inactivation of the MgrB regulator is not associated with decreased virulence of sequence type 258 KPC carbapenemase-producing Klebsiella pneumoniae. Antimicrob Agents Chemother 60:2509–2512. doi:10.1128/AAC.02981-15. 26824959 PMC4808163

[50] Adams MD, Bishop B, Wright MS 2016 Quantitative assessment of insertion sequence impact on bacterial genome architecture. Microb Genom 2:e000062. doi:10.1099/mgen.0.000062. 28348858 PMC5343135

[51] Her H-L, Wu Y-W 2018 A pan-genome-based machine learning approach for predicting antimicrobial resistance activities of the Escherichia coli strains. Bioinformatics 34:i89–i95. doi:10.1093/bioinformatics/bty276. 29949970 PMC6022653

[52] Kouchaki S, CRyPTIC Consortium, Yang Y, Walker TM, Walker AS, Wilson DJ, Peto TEA, Crook DW, Clifton DA, Consortium CR 2019 Application of machine learning techniques to tuberculosis drug resistance analysis. Bioinformatics 35:2276–2282. doi:10.1093/bioinformatics/bty949. 30462147 PMC6596891

[53] Yang Y, Niehaus KE, Walker TM, Iqbal Z, Walker AS, Wilson DJ, Peto TEA, Crook DW, Smith EG, Zhu T, Clifton DA 2018 Machine learning for classifying tuberculosis drug-resistance from DNA sequencing data. Bioinformatics 34:1666–1671. doi:10.1093/bioinformatics/btx801. 29240876 PMC5946815

[54] Jeni LA, Cohn JF, De La Torre F 2013 Facing imbalanced data recommendations for the use of performance metrics. Int Conf Affect Comput Intell Interact Workshops 2013:245–251. doi:10.1109/ACII.2013.47. 25574450 PMC4285355

[55] Holt KE, Wertheim H, Zadoks RN, Baker S, Whitehouse CA, Dance D, Jenney A, Connor TR, Hsu LY, Severin J, Brisse S, Cao H, Wilksch J, Gorrie C, Schultz MB, Edwards DJ, Nguyen KV, Nguyen TV, Dao TT, Mensink M, Minh VL, Nhu NT, Schultsz C, Kuntaman K, Newton PN, Moore CE, Strugnell RA, Thomson NR 2015 Genomic analysis of diversity, population structure, virulence, and antimicrobial resistance in Klebsiella pneumoniae, an urgent threat to public health. Proc Natl Acad Sci U S A 112:E3574–81. doi:10.1073/pnas.1501049112. 26100894 PMC4500264

[56] Read TD, Massey RC 2014 Characterizing the genetic basis of bacterial phenotypes using genome-wide association studies: a new direction for bacteriology. Genome Med 6:109. doi:10.1186/s13073-014-0109-z. 25593593 PMC4295408

[57] Clinical and Laboratory Standards Institute. 2015 Performance standards for antimicrobial susceptibility testing, 25th informational supplement. M100-S25. Clinical and Laboratory Standards Institute, Wayne, PA.

[58] Giddins MJ, Macesic N, Annavajhala MK, Stump S, Khan S, McConville TH, Mehta M, Gomez-Simmonds A, Uhlemann AC 2018 Successive emergence of ceftazidime-avibactam resistance through distinct genomic adaptations in blaKPC-2-harboring Klebsiella pneumoniae sequence type 307 isolates. Antimicrob Agents Chemother 62:e02101-17. doi:10.1128/AAC.02101-17. 29263067 PMC5826117

[59] Gomez-Simmonds A, Annavajhala MK, Wang Z, Macesic N, Hu Y, Giddins MJ, O’Malley A, Toussaint NC, Whittier S, Torres VJ, Uhlemann AC 2018 Genomic and geographic context for the evolution of high-risk carbapenem-resistant Enterobacter cloacae complex clones ST171 and ST78. mBio 9:e00542-18. doi:10.1128/mBio.00542-18. 29844109 PMC5974468

[60] Macesic N, Gomez-Simmonds A, Sullivan SB, Giddins MJ, Ferguson SA, Korakavi G, Leeds D, Park S, Shim K, Sowash MG, Hofbauer M, Finkel R, Hu Y, West J, Toussaint NC, Greendyke WG, Miko BA, Pereira MR, Whittier S, Verna EC, Uhlemann AC 2018 Genomic surveillance reveals diversity of multidrug-resistant organism colonization and infection: a prospective cohort study in liver transplant recipients. Clin Infect Dis 67:905–912. doi:10.1093/cid/ciy199. 29718144 PMC6117442

[61] European Committee on Antimicrobial Susceptibility Testing. 2016 Breakpoint tables for interpretation of MICs and zone diameters. http://www.eucast.org. Accessed 14 December 2018.

[62] Seemann T 2018 Shovill: faster SPAdes (or better SKESA/Megahit/Velvet) assembly of Illumina reads. https://github.com/tseemann/shovill. Accessed 23 July 2018.

[63] Holt KE 2019 Kleborate. https://github.com/katholt/Kleborate. Accessed 15 August 2019.

[64] Inouye M, Dashnow H, Raven LA, Schultz MB, Pope BJ, Tomita T, Zobel J, Holt KE 2014 SRST2: rapid genomic surveillance for public health and hospital microbiology labs. Genome Med 6:90. doi:10.1186/s13073-014-0090-6. 25422674 PMC4237778

[65] Wick RR, Judd LM, Gorrie CL, Holt KE 2017 Unicycler: resolving bacterial genome assemblies from short and long sequencing reads. PLoS Comput Biol 13:e1005595. doi:10.1371/journal.pcbi.1005595. 28594827 PMC5481147

[66] Seemann T 2018 Snippy: rapid haploid variant calling and core SNP phylogeny. https://github.com/tseemann/snippy. Accessed 23 July 2018.

[67] Earle SG, Wu CH, Charlesworth J, Stoesser N, Gordon NC, Walker TM, Spencer CCA, Iqbal Z, Clifton DA, Hopkins KL, Woodford N, Smith EG, Ismail N, Llewelyn MJ, Peto TE, Crook DW, McVean G, Walker AS, Wilson DJ 2016 Identifying lineage effects when controlling for population structure improves power in bacterial association studies. Nat Microbiol 1:16041. doi:10.1038/nmicrobiol.2016.41. 27572646 PMC5049680

[68] Lees JA, Vehkala M, Valimaki N, Harris SR, Chewapreecha C, Croucher NJ, Marttinen P, Davies MR, Steer AC, Tong SY, Honkela A, Parkhill J, Bentley SD, Corander J 2016 Sequence element enrichment analysis to determine the genetic basis of bacterial phenotypes. Nat Commun 7:12797. doi:10.1038/ncomms12797. 27633831 PMC5028413

[69] Jaillard M, Lima L, Tournoud M, Mahe P, van Belkum A, Lacroix V, Jacob L 2018 A fast and agnostic method for bacterial genome-wide association studies: bridging the gap between k-mers and genetic events. PLoS Genet 14:e1007758. doi:10.1371/journal.pgen.1007758. 30419019 PMC6258240

[70] Lees JA, Tien Mai T, Galardini M, Wheeler NE, Corander J 2019 Improved inference and prediction of bacterial genotype-phenotype associations using pangenome-spanning regressions. bioRxiv doi:10.1101/852426. PMC7343994 32636251

[71] Robin X, Turck N, Hainard A, Tiberti N, Lisacek F, Sanchez JC, Muller M 2011 pROC: an open-source package for R and S+ to analyze and compare ROC curves. BMC Bioinformatics 12:77. doi:10.1186/1471-2105-12-77. 21414208 PMC3068975

[72] Ramos PIP, Custódio MGF, Quispe Saji GDR, Cardoso T, da Silva GL, Braun G, Martins WMBS, Girardello R, de Vasconcelos ATR, Fernández E, Gales AC, Nicolás MF 2016 The polymyxin B-induced transcriptomic response of a clinical, multidrug-resistant Klebsiella pneumoniae involves multiple regulatory elements and intracellular targets. BMC Genomics 17:737. doi:10.1186/s12864-016-3070-y. 27801293 PMC5088521

[73] Brinkworth AJ, Hammer CH, Olano LR, Kobayashi SD, Chen L, Kreiswirth BN, DeLeo FR 2015 Identification of outer membrane and exoproteins of carbapenem-resistant multilocus sequence type 258 Klebsiella pneumoniae. PLoS One 10:e0123219. doi:10.1371/journal.pone.0123219. 25893665 PMC4404324

[74] Miethke M, Marahiel MA 2007 Siderophore-based iron acquisition and pathogen control. Microbiol Mol Biol Rev 71:413–451. doi:10.1128/MMBR.00012-07. 17804665 PMC2168645

[75] Bhagirath AY, Li Y, Patidar R, Yerex K, Ma X, Kumar A, Duan K 2019 Two component regulatory systems and antibiotic resistance in Gram-negative pathogens. Int J Mol Sci 20:E1781. doi:10.3390/ijms20071781. 30974906 PMC6480566

[76] Dorman MJ, Feltwell T, Goulding DA, Parkhill J, Short FL 2018 The capsule regulatory network of Klebsiella pneumoniae defined by density-TraDISort. mBio 9:e01863-18. doi:10.1128/mBio.01863-18. 30459193 PMC6247091

[77] Lamarche MG, Wanner BL, Crepin S, Harel J 2008 The phosphate regulon and bacterial virulence: a regulatory network connecting phosphate homeostasis and pathogenesis. FEMS Microbiol Rev 32:461–473. doi:10.1111/j.1574-6976.2008.00101.x. 18248418

[78] Nganje CN, Haynes SA, Qabar CM, Lent RC, Bou Ghanem EN, Shainheit MG 2019 PepN is a non-essential, cell wall-localized protein that contributes to neutrophil elastase-mediated killing of Streptococcus pneumoniae. PLoS One 14:e0211632. doi:10.1371/journal.pone.0211632. 30707714 PMC6358159

[79] Pitt ME, Cao MD, Butler MS, Ramu S, Ganesamoorthy D, Blaskovich MAT, Coin LJM, Cooper MA 2019 Octapeptin C4 and polymyxin resistance occur via distinct pathways in an epidemic XDR Klebsiella pneumoniae ST258 isolate. J Antimicrob Chemother 74:582–593. doi:10.1093/jac/dky458. 30445429 PMC6376851

[80] Hooper DC, Jacoby GA 2015 Mechanisms of drug resistance: quinolone resistance. Ann N Y Acad Sci 1354:12–31. doi:10.1111/nyas.12830. 26190223 PMC4626314

[81] Klein G, Raina S 2019 Regulated assembly of LPS, its structural alterations and cellular response to LPS defects. Int J Mol Sci 20:E356. doi:10.3390/ijms20020356. 30654491 PMC6358824

[82] Tong S, Lin Y, Lu S, Wang M, Bogdanov M, Zheng L 2016 Structural insight into substrate selection and catalysis of lipid phosphate phosphatase PgpB in the cell membrane. J Biol Chem 291:18342–18352. doi:10.1074/jbc.M116.737874. 27405756 PMC5000081

