# Supplementary figures and images for: Predicting Phenotypic Polymyxin Resistance in Klebsiella pneumoniae through Machine Learning Analysis of Genomic Data
Source: mSystems. 2020 May 26;5(3):e00656-19. doi: 10.1128/mSystems.00656-19 (PMC7253370; doi:10.1128/mSystems.00656-19)

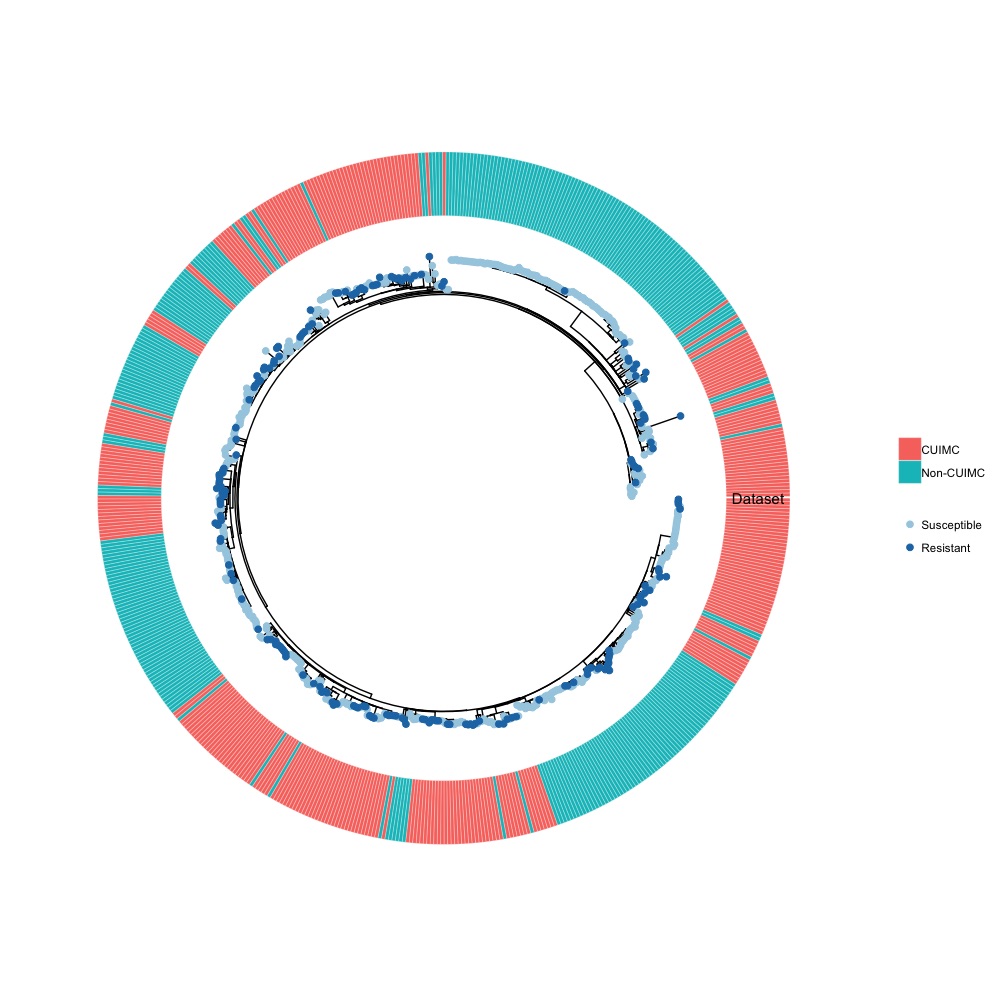

Supplement: FIG S1 [file mSystems.00656-19-sf001.jpg]
